# Prognostic indicators associated with 2-week survival and postoperative antimicrobial use following ovariohysterectomy for canine pyometra from 2012 to 2021: a multicenter retrospective cohort of 470 cases

**DOI:** 10.3389/fvets.2026.1935847

**Published:** 2026-09-22

**Authors:** Sarah M. Langfitt, Aparna Arun, Daniel I. Spector, Freddy J. Maza, Sophia Buoniconti, Penny J. Regier, Morgan E. Hardie, Sarah Marvel, Janet A. Grimes

**Affiliations:** 1 Department of Surgery, BluePearl Pet Hospital, Levittown, PA, United States; 2 Hospital for Veterinary Surgery, New York, NY, United States; 3 Department of Surgery, Schwarzman Animal Medical Center, New York, NY, United States; 4 Wayside Waifs, Kansas City, MO, United States; 5 College of Veterinary Medicine, University of Florida, Gainesville, FL, United States; 6 School of Veterinary Medicine, University of California, Davis, CA, United States; 7 College of Veterinary Medicine and Biomedical Sciences, Colorado State University, Fort Collins, CO, United States; 8 Department of Small Animal Medicine and Surgery, College of Veterinary Medicine, University of Athens, Athens, GA, United States

**Keywords:** antimicrobial resist ance, antimicrobial stewardship (AMS), prognosis, pyometra, retrospective

## Abstract

**Objective:**

This study aimed to retrospectively evaluate factors associated with 2-week survival following ovariohysterectomy for pyometra in dogs.

**Animals:**

This study included 470 intact bitches that underwent ovariohysterectomy for pyometra.

**Methods:**

In this multi-institutional retrospective cohort study, medical records were reviewed for clinical data, including signalment, duration of clinical signs, blood count findings, presence of septic peritonitis, culture and susceptibility results, antibiotics prescribed and duration, and survival at 2 weeks postoperatively (from the medical record or via owner contact). A univariable analysis was performed due to the low mortality rate to evaluate the association between variables and 2-week survival.

**Results:**

Four hundred and fifty-two dogs survived to discharge, of which 10 dogs (2.2%) did not survive the 2-week follow-up. Eighteen dogs (3.8%) did not survive to discharge (DNSD). Post-discharge 2-week survival was 97.8% (442/452) and was associated with younger age and the absence of pre-operative azotemia, hyperkalemia, and hypoglycemia (*p*-values: 0.005, 0.043, 0.004, and 0.015, respectively). No association was found with pyometra type (open vs. closed), oral antibiotic use or duration, or any other assessed variable (*p*-values > 0.05).

**Clinical significance:**

Younger dogs that were presented for pyometra without azotemia, hyperkalemia, and/or hypoglycemia had improved 2-week postoperative survival. An association between postoperative discharge antibiotic use and 2-week survival was not identified in this study. Further studies with greater statistical power are needed to evaluate the potential effect of postoperative antibiotic use on survival.

## Introduction

Pyometra, an infection of the uterus, is associated with a lifetime risk of 15%–24% in intact bitches and may be open (with active drainage of purulent material) or closed (without active drainage of purulent material) (1–3). *Escherichia coli* is the most frequently isolated bacterium from uterine purulent exudate in canines with pyometra (4, 5). The pathophysiology of canine pyometra is multifactorial and has been previously studied in relation to the roles of reproductive hormones and *E. coli* virulence factors (6, 7). Bacterial cystitis was present in 33% of pyometra cases in one study, and the same isolates of *E. coli* were identified in urine and uterine samples from individual dogs. This suggests a relationship between the bacterial populations at these two sites but does not establish a causal relationship (8). In canine bacterial cystitis, where source control cannot be achieved, antimicrobial stewardship guidelines now recommend using culture and susceptibility results to guide subsequent antimicrobial selection and changing empiric therapy when the isolated organism is resistant to the initial antimicrobial, providing a framework for considering whether similar susceptibility-directed adjustments may be warranted in dogs with pyometra (9). The virulence factors of *E. coli* facilitate uterine infection by promoting bacterial adhesion, invasion of endometrial tissue, and biofilm formation (10, 11). The presence of *E. coli* α-hemolysin (HlyA) damages tissues and compromises the early uterine immune response (6). Ovariectomy and ovariohysterectomy remain the most reliable measures to prevent the development of pyometra (12, 13). Medical management of canine pyometra, including the use of aglepristone with or without cloprostenol, has varying success, with reports of recurrence in 19% of monitored bitches (14–17).

When a canine patient with pyometra is presented to a veterinary hospital, initial pre-operative treatments generally include fluid resuscitation and administration of antibiotics prior to surgical intervention. These treatments often vary significantly due to the broad spectrum of clinical presentations encountered by clinicians. The definitive treatment of choice for canine pyometra remains ovariohysterectomy, as it eliminates the reservoir of infection (1, 7, 18–20). Following surgery, dogs are often treated with intravenous fluids, analgesics, and antibiotics. Although antibiotic therapy is commonly used, the clinical benefits of postoperative antibiotic therapy across the spectrum of disease severity remain unknown. Previous studies have reported favorable outcomes despite discrepancies between bacterial susceptibility and prescribed antibiotic therapy (21). The survival rate with surgical management of pyometra is historically very high (97%) across different treatment settings, including non-specialized and community medicine practices (22, 23). Despite this high survival rate, factors associated with survival across the spectrum of disease and the use of antibiotic therapy remain incompletely described. This study aimed to retrospectively evaluate factors associated with 2-week survival following ovariohysterectomy for pyometra in dogs.

## Materials and methods

The medical record databases at the Schwarzman Animal Medical Center, the University of California Davis, the University of Florida, the University of Georgia, and Colorado State University were searched by multiple investigators for canine patients who underwent ovariohysterectomy for pyometra from 2012 to 2021. The data were then aggregated by a single reviewer (AA). Dogs were included if they underwent ovariohysterectomy at the presenting institution to treat pyometra. Dogs were excluded if they were pregnant; if a diagnosis of pyometra could not be confirmed based on a description of vulvar discharge or uterine fluid (purulent/mucopurulent/hemopurulent) or postoperative testing (positive culture or consistent histopathology); if the medical record was incomplete; or if it could not be established whether the patient was alive 2 weeks postoperatively.

Data collected from the medical records included history (duration of clinical signs and the presence of vulvar discharge), signalment, vitals, physical exam findings (whether there was active vulvar discharge), point-of-care and select pre-operative comprehensive blood testing results at the time of admission (packed cell volume, total solids, potassium, glucose, lactate, presence of neutrophilia or neutropenia, and presence of azotemia), and peri-operative injectable antibiotic and dose (from the completed anesthesia protocol as well as the postoperative record); whether the patient had septic peritonitis (based on the surgery report); whether the uterine or intra-abdominal contents were cultured, culture and susceptibility results; whether an antibiotic was chosen for oral therapy postoperatively to which the cultured organism was susceptible, the dose and duration of the oral antibiotic, and the same information for each additional oral antibiotic (if prescribed), and whether the patient was alive 2 weeks postoperatively. If review of the medical record was insufficient to determine survival at 2 weeks postoperatively, owners were contacted by phone or email and asked up to four questions based on their responses—whether their pet was alive currently, when their pet passed, if the cause of death was known, and what the cause of death was. From this information, it was determined whether the patient was alive 2 weeks postoperatively. Cases were classified based on survival: patients that did not survive to discharge (DNSD), patients that did not survive to 2 weeks following discharge (DNS2W), and patients that were alive at 2 weeks (S2W).

Neutropenia was defined as a neutrophil count of less than 3,000/μL, while neutrophilia was defined as a neutrophil count of greater than 13,000/μL based on reference laboratory ranges. Similarly, hypoalbuminemia was defined as an albumin concentration of less than 2.7 mg/dL, and azotemia was defined as a creatinine concentration of greater than 1.5 mg/dL. The duration of postoperative antibiotics was classified into three categories: 1–7 days, 8–14 days, and >14 days. Due to the retrospective nature of the study, information about a change in antibiotics based on culture and susceptibility results was not always available; hence, this information was not collected.

### Statistical analysis

A statistical analysis was performed using NCSS 2023 (Kaysville, UT, USA). Data variables were classified as continuous, categorical, or binary variables. Due to the low number of post-discharge non-survivors at 2 weeks postoperatively (10), multivariable statistical analysis was not possible, and the associations between factors and post-discharge 2-week survival were assessed by means of univariable statistics. Chi-squared tests were used for binary and categorical data, while unpaired t-tests were used for continuous data. Fisher’s exact test was used instead of the chi-squared test when the expected count in a cell of the chi-square was less than 5. The Wilcoxon rank sum test was used instead of a t-test when the assumption of normality (Shapiro–Wilk test) and/or homogeneity of variance was violated. The level of significance was set at a *p*-value of < 0.05 for all comparisons between post-discharge groups.

When comparing the three groups (DNSD, DNS2W, and S2W), if the residuals were normal and the variances were homogeneous, the parametric one-way analysis of variance (ANOVA) was used. When residuals were not normal and/or variances were heterogeneous, the Kruskal–Wallis test was used. Normality of the residuals was assessed using a histogram and normal probability plot. Homogeneity of variance was assessed using a Bartlett test. Following analysis, if a significant difference among the three groups was identified, *post-hoc* testing was performed using Tukey’s test (ANOVA) or Dunn’s test (Kruskal–Wallis test). Chi-squared tests were used for binary and categorical data, while unpaired t-tests were used for continuous data. Fisher’s exact test was used instead of the chi-squared test when the expected count in a cell of the chi-square was less than 5. When making two comparisons among the three groups, the cutoff *p*-value was 0.025, and when making all three comparisons between the three groups, the cutoff *p*-value was 0.017. Normal data were reported as mean and standard deviation, while non-normal data were reported as median and 10th/90th percentiles.

## Results

Eight hundred and thirty-three dogs were identified from the five institutions. Three hundred and sixty-three dogs were excluded: 203 for an inability to confirm a diagnosis of pyometra, 141 for lack of a 2-week follow-up, 16 for incomplete medical records, 2 because they were found to be pregnant, and 1 because a perforated duodenal ulcer was found at the time of surgery, and the patient subsequently died the next day. Four hundred and seventy patients met the inclusion criteria.

As there were too many breeds (101) to draw a meaningful comparison, breeds were removed from the analysis. Clinical signs included a variable combination of lethargy, hyporexia, vomiting, and vaginal discharge. The mean age was 8 ± 3.3 years. The mean age of patients who did not survive to be discharged was 8.9 ± 3.5 years. The mean age of post-discharge non-survivors was 10.7 ± 2.6 years compared to 7.9 ± 3.3 years for survivors. Post-discharge 2-week survival was associated with age (*p* = 0.005), with an increase in the probability of non-survival with increasing age. When analyzing age between DNSD, DNS2W, and S2W, a statistically significant difference was identified (*p* = 0.01), and *post-hoc* testing showed that DNS2W was significantly different from S2W.

Closed pyometra was diagnosed in 30.4% (143) of dogs, while open pyometra was diagnosed in 69.6% (327) of dogs. Clinical signs were present for a median of 3 days (range 0–180 days) prior to presentation. The median weight was 13 kg (range 1.2–86 kg). On admission, the median temperature was 101.7 °F (range 94.3–107.2 °F), the median heart rate was 136 beats/min (range 50–249 beats/min), and the median respiratory rate was 36 breaths/min (range 18–100 breaths/min) at presentation. There was no statistically significant difference in open vs. closed pyometra (*p* = 0.294), duration of clinical signs prior to presentation (*p* = 0.082), weight (*p* = 0.461), temperature at presentation (*p* = 0.599), heart rate at presentation (*p* = 0.343), and respiratory rate at presentation (*p* = 0.658) between DNS2W and S2W.

There was no association between the three survival groups and whether patients presented with open or closed pyometra (*p* = 0.18). When analyzing presenting temperature between DNSD (mean 100.23, SD 1.89), DNS2W (mean 101.21, SD 2.49), and S2W (mean 101.76, SD 1.61), there was a statistically significant difference (*p* < 0.0001), with DNSD dogs having significantly lower temperatures than S2W. There was no statistically significant association (*p* = 0.22) when analyzing the duration of clinical signs prior to presentation between DNSD (median 3, 0.9–35.6), DNS2W (median 7, 1.1–83.1), and S2W (median 3, 1–14). There was no statistically significant association (*p* = 0.06) when analyzing weight between DNSD (median 5.1, 2.1–31.8), DNS2W (median 13.7, 1.5–53.6), and S2W (median 13.2, 2.9–38.6). There was nothing significant on *post-hoc* analyses either. There was no statistically significant association (*p* = 0.82) when analyzing heart rate at presentation between DNSD (mean 134.56, SD 22.42), DNS2W (mean 141.20, SD 29.5), and S2W (mean 135.4, SD 29.4). There was no statistically significant association (*p* = 0.06) when analyzing respiratory rate at presentation between DNSD (median 40, 26.4–55.2), DNS2W (median 40, 24–70), and S2W (median 36, 24–50).

The median packed cell volume was 42% (range 12–67%), the median total solids was 8 g/dL (range 3.8–13.9 g/dL), the median potassium concentration was 4 mmol/L (range 2.6–6.9 mmol/L), the median glucose concentration was 98 mg/dL (range 32–429 mg/dL), and the median lactate concentration was 2.4 mmol/L (range 0.5–28 mmol/L). Neutrophil count was reported for 384 dogs; 59.9% (230) had neutrophilia, and 8.6% (33) had neutropenia. Albumin concentration was reported in 360 dogs, and 55.3% (199) had hypoalbuminemia. Creatinine concentration was reported in 429 dogs, and 15.6% (67) had azotemia. A septic abdomen was diagnosed in 7.4% (35) of dogs, and one of these dogs was noted to have a perforated pyloric ulcer, while the other cases were suspected to be secondary to pyometra, as another source of sepsis was not identified. Seven of these dogs did not survive to be discharged. All 28 dogs with a septic abdomen that were discharged were noted to be alive 2 weeks after surgery. Only one of these dogs was not given oral antibiotics upon discharge.

There was no statistically significant association (*p* = 0.30) when analyzing packed cell volume at presentation between DNSD (mean 40.8, SD 11.8), DNS2W (mean 36.8, SD 9.6), and S2W (mean 41, SD 8.4). There was no statistically significant association (*p* = 0.26) between total solids at presentation in DNSD (mean 7.2, SD 2.3), DNS2W (mean 8.4, SD 1.3), and S2W (mean 8, SD 1.3). There was no statistically significant association (*p* = 0.3) when analyzing lactate at presentation between DNSD (median 3.2, 0.7–9.2), DNS2W (median 2.9, 1.5–7), and S2W (median 2.4, 1.2–4.6). When analyzing potassium levels between DNSD (median 4.3, 3.1–6.1), DNS2W (median 4.6, 3.99–6.4), and S2W (median 4, 3.4–4.9), potassium levels differed significantly (*p* = 0.01), with DNSD having higher potassium levels than S2W. When analyzing glucose levels between DNSD (median 92, 43.3–109.7), DNS2W (median 80, 61–107), and S2W (median 98, 70–130.2), glucose levels differed significantly (*p* = 0.004), with DNSD and DNS2W being statistically different from S2W.

There was no association between the three groups and the presence of neutrophilia (*p* = 0.25), neutropenia (*p* = 0.07), and hypoalbuminemia (*p* = 0.11). Azotemia differed significantly between the groups (*p* = 0.002), where 13.9% of the dogs that survived had azotemia, while 40% of those dogs that died had azotemia. The presence of a septic abdomen also differed significantly between the groups (*p* = 0.0002), where 38.9% of the dogs that died before discharge had a septic abdomen compared to 6.3% of survivors.

Peri-operative antibiotics were used in 99.6% (468) of cases. Reasons for not using antibiotics were not noted in the remaining two cases. Two different antibiotics were documented during hospitalization in 34.4% (161) of cases, and three different antibiotics were documented during hospitalization in 8.1% (38) of cases. This included cases where an oral antibiotic had been started by a primary care veterinarian prior to presentation. Peri-operative antibiotics used included ampicillin-sulbactam (405), enrofloxacin (93), cefazolin (82), cefoxitin (31), amoxicillin-clavulanate (9), ampicillin (8), metronidazole (5), cefovecin (4), amoxicillin (2), ticarcillin-clavulanate (1), clindamycin (1), ciprofloxacin (1), and cefpodoxime (1). Based on the recommended doses, an appropriate dose was administered in 77.7% (518) of instances of antibiotic prescription, a lower dose than the recommended range was administered in 2.7% (18) of antibiotic prescriptions, and a higher dose than the recommended range was administered in 8.5% (57) of antibiotic prescriptions (1, 24). Susceptibility or resistance of cultured bacteria was reported specifically for oral postoperative antibiotics.

Bacterial culture and susceptibility of uterine fluid or peritoneal effusion (for septic abdomen cases) were performed in 55.1% (259) of dogs, of which 92.3% (239) had a positive culture. A total of 28.9% (69/229) of positive cultures were polymicrobial infections. *E. coli* was the most frequently cultured organism, with 67.8% (162) of positive cultures having *E. coli* as the first organism cultured. Other frequently cultured bacteria included *Streptococcus* species (27), *Staphylococcus* species (19), unspecified enteric Gram-negative bacilli (13), *Pasteurella* species (10), *Enterococcus* species (9), and *Proteus* species (7). No growth was noted in 7.7% (20) of cases that were diagnosed with pyometra, either by noting active discharge (15/20) at the time of presentation, noting purulent uterine fluid intra-operatively (3/20), or noting bacteria on histopathology (2/20).

Oral antibiotics were prescribed postoperatively in 91.8% (415) of dogs that survived to be discharged. Two oral antibiotics were prescribed in 21.4% (89) of cases. Amoxicillin-clavulanate was the most frequently prescribed oral antibiotic (92.5%, 384), followed by enrofloxacin (21.4%, 89). Other oral antibiotics included cefpodoxime (3.6%, 15), metronidazole (1.2%, 5), doxycycline (0.7%, 3), cephalexin (0.5%, 2), marbofloxacin (0.2%, 1), ciprofloxacin (0.2%, 1), clindamycin (0.2%, 1), and ormetoprim-sulfadimethoxine (0.2%, 1). Based on the recommended doses, an appropriate dose was prescribed in 89.7% (452) of antibiotic prescriptions, a dose lower than the recommended range was prescribed in 6.3% (32) of cases, and a dose higher than the recommended range was prescribed in 2.6% (13) of cases (1). Postoperative oral antibiotics were prescribed for a duration of 1–7 days (140/413, 33.9%), 8–14 days (263/413, 63.7%), and >14 days (10/413, 2.4%); for two dogs, the duration of the postoperative antibiotic course was not reported. The rationale for prescribing or not prescribing antibiotics was not noted.

Ten dogs with a positive culture did not survive to be discharged. Among dogs that survived to discharge, an oral antibiotic to which the cultured bacteria were susceptible was prescribed in 50.2% (115/229) of cases, whereas an oral antibiotic to which the cultured bacteria were resistant was initially prescribed in 26.2% (60/229) of cases. There was no statistical significance between whether a susceptible or resistant antibiotic had been prescribed and 2-week survival (*p* = 0.55); however, among the 10 cases that died following discharge, only 3 were prescribed antibiotics to which the bacteria were susceptible, 2 had no growth on culture, 4 had no culture performed, and 1 was not prescribed an oral antibiotic. For the remaining cases, either the antibiotic was of intermediate susceptibility (5/229), no susceptibility was performed (23/229), the antibiotic was not on the susceptibility panel (4/229), no antibiotic was prescribed (13/229), or the prescribed antibiotic was listed as not indicated (9/229) on the susceptibility report.

*Escherichia coli,* specifically, was susceptible to the empirically chosen antibiotic in 53.9% (82/152) of dogs, intermediate in 2.6% (4/152) of dogs, and resistant in 30.9% (47/152) of dogs. Antibiotics were not prescribed in 7.2% (11/152) of dogs, or the prescribed antibiotic was not on the reported sensitivity panel in 5.3% (8/152) of dogs.

Eighteen dogs did not survive to be discharged. Eleven dogs experienced cardiopulmonary arrest, while the remaining seven were euthanized due to declining conditions. While comorbidities were not specifically collected in this study, of the 11 dogs that experienced cardiac arrest postoperatively, 1 had a concurrent mammary gland tumor removed, 1 dog had a diffuse bronchointerstitial pattern noted at the time of its decompensation, one dog’s status was post-return to spontaneous circulation (having arrested once preoperatively and been successfully resuscitated) when it experienced a second cardiopulmonary arrest post-operatively. Of the seven dogs that were euthanized, two were described as septic, one was euthanized due to a metastatic pancreatic islet cell tumor discovered at the time of surgery, one had a gastrointestinal stromal cell tumor and developed necrotizing pancreatitis, one developed a suspected thromboembolism causing acute decompensation, and two were euthanized due to progressive postoperative clinical decline that was not further classified. Once discharged, survival at 2 weeks postoperatively was 97.8% (442/452). Of the 10 dogs that did not survive post-discharge, 4 were euthanized, 4 died suddenly at home, 1 was re-presented 1 week postoperatively with a septic abdomen and experienced cardiopulmonary arrest 1 day following repeat exploratory laparotomy, which noted an abscess at the uterine stump, and 1 had a concurrent cavotomy for thrombectomy at the time of ovariohysterectomy and experienced cardiopulmonary arrest at 12 days postoperatively. Vital signs at the time of presentation were collected, but these variables were not collected uniformly across all cases and throughout hospitalization; therefore, they cannot be reliably used to characterize the initial severity of illness between survivors and non-survivors.

Two-week survival was negatively associated with the presence of pre-operative azotemia (*p* = 0.043), pre-operative hyperkalemia (*p* = 0.004), and pre-operative hypoglycemia (*p* = 0.015). There was no statistically significant association between 2-week survival and pre-operative PCV (*p* = 0.224), pre-operative TS (*p* = 0.575), presence of neutrophilia (*p* = 1.000), presence of neutropenia (*p* = 0.486), presence of pre-operative hypoalbuminemia (*p* = 0.077), presence of pre-operative hyperlactatemia (*p* = 0.279), presence of septic abdomen (*p* = 1.000), whether an oral antibiotic was prescribed postoperatively (*p* = 0.578), or duration of postoperative oral antibiotic course (*p* = 0.567). Azotemia and potassium concentrations were related. Azotemic patients were noted to have a mean potassium level of 4.5 mEq/L (standard deviation 0.78), while non-azotemic patients were noted to have a mean potassium level of 4.06 mEq/L (standard deviation 0.59). When comparing only DNSD with S2W, statistical significance was noted with the presence of neutropenia (*p* = 0.05), azotemia (*p* = 0.007), and septic abdomen (*p* = 0.0001), while no statistical significance was noted with open vs. closed pyometra (*p* = 0.20), presence of neutrophilia (*p* = 0.1), and presence of hypoalbuminemia (*p* = 0.34).

## Discussion

Increasing age and the presence of azotemia, hyperkalemia, and/or hypoglycemia were associated with post-discharge mortality at 2 weeks postoperatively in this study. No significant difference was detected between 2-week postoperative survival and pyometra type (open vs. closed), duration of clinical signs, vital signs at presentation, specific pre-operative blood work findings (PCV, TS, neutrophilia, neutropenia, hypoalbuminemia, and hyperlactatemia), presence of septic peritonitis at the time of surgery, or postoperative oral antibiotic use or duration in this study. Survival following ovariohysterectomy for pyometra in this study (97.8%) was consistent with previously reported survival rates of 97% (23, 25). The lack of statistical significance regarding the duration of clinical signs prior to presentation (*p* = 0.082) may be due to a type II statistical error from the small number of 2-week non-survivors.

An older age at the time of presentation was associated with decreased survival. This may be due to the presence of comorbidities that affect peri-operative mortality; however, information about comorbidities was not collected for this study. Azotemia has also previously been shown to be associated with mortality following ovariohysterectomy for pyometra (26). Renal dysfunction, with confirmed histopathologic changes, has been shown to be secondary to pyometra, where dogs with higher renal parameters and urinary biomarkers were more likely to experience acute renal failure (27, 28). In this study, azotemic patients also had a higher potassium concentration. Hyperkalemic dogs are more likely to experience mortality compared to normokalemic dogs, and this may be due to hyperkalemia-associated arrhythmia or may reflect the severity of systemic disease (29).

Non-diabetic hypoglycemia has also been associated with sepsis, gastrointestinal disease, and neoplasia, with hypoglycemic dogs having a higher mortality rate than euglycemic dogs in an emergency setting (30). No specific studies have looked at the effect of hypoglycemia on mortality in dogs with pyometra. Prior studies have documented more severe illness, electrocardiographic (ECG) abnormalities, arrhythmia, and toxic shock-like syndrome in dogs with closed pyometra (31–34); however, those studies, similar to our findings, did not report a difference in 2-week survival between bitches that were presented with open vs. closed pyometra. Severity of illness between open and closed pyometra in this cohort cannot be determined because information regarding the duration of hospitalization, ECG findings, and severity of illness was not always available and hence was not collected. Vital signs at the time of presentation were collected, but variables such as blood pressure or indicators of SIRS postoperatively were not collected uniformly across all cases and throughout hospitalization; therefore, they cannot be reliably used to characterize the initial severity of illness between survivors and non-survivors.

In a recent survey of Arizona-licensed veterinarians, 74% stated that they used postoperative antibiotics for the treatment of pyometra, while only 12% regularly submitted samples for bacterial culture and antibiotic susceptibility (25). In this study, only 50.2% of the cultured organisms were susceptible to the prescribed antibiotics, and 26.2% of cultured organisms were resistant to the prescribed antibiotics. There was no statistically significant difference in survival times between patients who were prescribed antibiotics and those who were not. The high rates of antimicrobial use in the pre- and postoperative periods and the low number of non-survivors limit the power of the statistical analysis. *E. coli* was the most common bacterium isolated in the dogs in this study, which is consistent with previous literature (4, 5, 10, 35). Despite its predominance, *E. coli* was susceptible to the empirically selected antimicrobial in only 53.9% of cases. The most frequently isolated bacterium from pyometra, *E. coli*, has developed a more uniform resistance pattern to potentiated penicillins (18). Specifically, Enterobacterales, the order of bacteria to which *E. coli* belongs, are interpreted to be resistant to ampicillin, amoxicillin, and amoxicillin-clavulanate, as drug concentrations achieved from recommended doses are not high enough to reach the therapeutic target (36). This contrasts with other studies, which cultured populations of *E. coli* and other *Enterobacterales* species that were 93% susceptible to extended-spectrum beta-lactamases (ESBL) and 54% susceptible to penicillins (8). The differences in susceptibility patterns between the present US study and previous studies conducted in Finland may reflect geographic variation in antimicrobial susceptibility, although differences in study populations and methodology may also contribute. These findings highlight the importance of local surveillance to characterize regional susceptibility patterns and inform antimicrobial selection.

The predominance of ESBL-resistant *E. coli* in our study suggests that empiric antibiotic prescribing practices may be considered discordant with subsequent *in vitro* susceptibility data in a substantial proportion of cases. While this discordance does not indicate treatment failure, it highlights the limitations of relying on empiric antimicrobial selection in this population and represents an important target for antimicrobial stewardship practices. As exposure of bacteria, pathogenic or commensal, to antibiotics applies selection pressure that can result in the emergence or propagation of resistant populations, it is important to eliminate inappropriate use of antibiotics (37, 38). Multi-drug resistance (MDR) is an acquired non-susceptibility to at least one antimicrobial agent in three or more antimicrobial classes (39, 40). While MDR status was not collected in this study and the prevalence of MDR phenotypes cannot be estimated, the isolated *E. coli* was resistant to the prescribed empirical antibiotic in 30.9% of dogs, and MDR has been demonstrated in 24%–50% of *E. coli* isolates from dogs with pyometra (18, 41). Antimicrobial resistance among bacteria associated with pyometra may have broader One Health implications, given shared resistant bacterial clones and resistance genes documented in pathogens affecting both human and animal populations, potentially leading to treatment failure (37, 42, 43).

Similar positive outcomes despite discrepancies between susceptibility data and the prescribed antibiotic are described in 51 bitches treated for pyometra with ovariohysterectomy (21). Our findings extend these observations by demonstrating that discrepancies between empiric antimicrobial selection and subsequent susceptibility results are common in a larger clinical population, including dogs with septic abdominal disease, and that positive outcomes are observed in the majority of cases. Furthermore, Turkki et al. evaluated peri-operative antibiotic administration according to Swedish treatment guidelines and reported that approximately half of dogs recovered without antimicrobial treatment. They concluded that further studies are needed to identify which dogs benefit from antimicrobial therapy. Their findings highlight the ongoing uncertainty regarding which dogs require antibiotic therapy following surgical source control.

The results of our data and those from the cited studies highlight the importance of source control and raise an interesting question regarding septic peritonitis in pyometra: Is concordance between empiric antimicrobial therapy and susceptibility data more clinically important where the degree of source control is less definitive? This represents an important area for future research. More recently, a double-blinded randomized controlled trial of 152 dogs with uncomplicated pyometra demonstrated that placebo was non-inferior to a 5-day postoperative course of sulfadiazine-trimethoprim (8). These findings provide important evidence supporting judicious prescribing of antibiotics in dogs with uncomplicated pyometra; however, they do not address whether antimicrobial treatment influences survival and other clinically important outcomes in dogs with more severe disease. The present study describes antimicrobial prescribing at hospital discharge across a broad spectrum of disease severity, including dogs with septic processes. In the face of these findings, prospective studies with larger populations that compare antimicrobial strategies and evaluate clinically important outcomes in dogs with varying degrees of disease severity are warranted. This study may provide a useful framework for designing such prospective studies, including approaches to categorizing disease severity and evaluating antimicrobial strategies across different clinical presentations.

This study’s limitations include those inherent to its retrospective nature, which did not allow for all data points to be collected from all cases. The focus of this study was antibiotic prescriptions at hospital discharge; it did not differentiate between antibiotics administered pre-operatively and postoperatively while the patients were hospitalized or account for the duration of hospitalization. Therefore, total postoperative antibiotic exposure could not be determined, limiting our ability to draw conclusions about the outcomes related to total postoperative antimicrobial use. Due to the multi-institutional nature of the study, medical record searches were not uniform. Methods of clinical stabilization, hospitalization criteria, timing of surgery, surgical technique, volume of abdominal lavage, and discharge criteria were also not standardized. There was no statistically significant difference in survival times between patients who were prescribed antibiotics and those that were not. Interpretation of this finding is limited by the retrospective and non-randomized nature of antimicrobial treatment decisions, which were made according to the clinical judgment of the attending veterinarian. Furthermore, dogs receiving antimicrobial therapy may have differed systematically in disease severity from those that did not receive therapy, introducing potential confounding indications. Given the high prevalence of antimicrobial use in this population and the low number of non-survivors, the study had limited power to detect an association between antimicrobial treatment and survival. The exclusion of 141 dogs for which a 2-week follow-up was unavailable represents a potential source of selection bias. As these dogs did not meet the predefined inclusion criteria, additional data were not collected, precluding comparison with the study’s population; therefore, the magnitude and direction of any potential selection bias cannot be determined. Due to the low mortality rate, a multivariable model could not be constructed, and only univariable associations could be evaluated; therefore, individual factors associated with outcome should not be interpreted as independent determinants of prognosis, as their associations may reflect a constellation of factors collectively indicative of disease severity. The number of individual comparisons introduces the possibility of Type I error. Readers are encouraged to analyze the univariate results (Table 1) to determine the clinical relevance of these findings.

**Table 1:** Difference in continuous variables between survivors and non-survivors.

Variable	Survivors	Cases that did not survive to discharge	2-week non-survivors discharged from the hospital
Postoperative oral antimicrobial usage	415/452 (91.8%)	N/A	9/10 (90%)
Age (years)	8 (0.5–17)*	9.5 (3–14)	10.6 (5–15)
Duration of clinical signs (days)	3 (0–180)	3 (0.21–41)	7 (1–90)
Weight (kilograms)	13.2 (1.2–86)	5.1 (1.3–33.5)	13.7 (1.5–55.4)
Temperature (Fahrenheit)	101.8 (94.3–107.2)*	100.7 (96–102.7)	101.4 (95.1–104)
Heart rate (beats/min)	136 (50–249)	138 (100–168)	145 (80–180)
Respiratory rate (breaths/min)	36 (18–100)	40 (24–60)	40 (24–70)
Packed cell volume (%)	42 (12–67)	42.3 (14–59)	36 (18–50)
Total solids (g/dL)	8 (3.8–13.9)	6.8 (4–11)	8.2 (7–11)
Potassium (mmol/L)	4 (2.6–6.9)*	4.3 (3–6.8)	4.6 (4–6.5)
Glucose (mg/dL)	98 (32–429)*	92 (37–116)	80 (61–107)
Lactate (mmol/L)	2.4 (0.5–28)	3.2 (0.6–14.1)	2.9 (1.5–7)

Continuous variables are reported as median (range); antimicrobial therapy binary variables are reported as number positive/total (%). All patients who did not survive to discharge died within 36 h of surgery and were not transitioned to oral antibiotics. Values marked with an asterisk were significantly correlated with survival (*p* < 0.05).

*En bloc* removal of an intact infected uterus results in removal of the reservoir of infection. For bitches with pyometra complicated by uterine rupture and septic peritonitis, infection extends beyond the uterus, and antimicrobial management may therefore differ from that of uncomplicated disease. In this study, no association between prescription of oral antibiotics at hospital discharge and 2-week survival was identified among dogs with pyometra and septic peritonitis. This finding should not be interpreted as evidence that postoperative antimicrobial therapy does not influence outcome, as the study had limited statistical power and did not account for antimicrobial treatment received during hospitalization, duration of therapy, or total antimicrobial exposure.

This may be because peritoneal lavage combined with peri-operative antibiotic doses was sufficient to treat the infection. It is also possible that these patients were hospitalized for a longer period compared to the rest of the study population and experienced a longer duration of intravenous antibiotic therapy; because this paper focused on antibiotics at discharge rather than inpatient use, the total antibiotic exposure in this population was likely underestimated.

The findings in this study show that azotemia, hypoglycemia, and hyperkalemia are negative prognostic indicators for patients with suspected pyometra. Overall survival was high in this study, although the relationship between survival and adequacy of source control could not be independently evaluated. Due to the low rate of non-survivors, the impact of postoperative antibiotics on survival is inconclusive. This represents a potential opportunity for a prospective, randomized study to evaluate the association between post-ovariohysterectomy antibiotic use and survival in dogs.

## Data Availability

The raw data supporting the conclusions of this article will be made available by the authors, without undue reservation.
